# Chest wall mechanics before and after diaphragm plication

**DOI:** 10.1186/s13019-016-0419-x

**Published:** 2016-02-02

**Authors:** Ghazi Elshafie, Johanna Acosta, Andrea Aliverti, Amy Bradley, Prem Kumar, Pala Rajesh, Babu Naidu

**Affiliations:** Heart of England Foundation Trust, Birmingham, B9 5SS UK; International Digital Laboratory, University of Warwick, Coventry, CV4 7AL UK; Dipartimento di Elettronica, Informazione e Bioingegneria, Politecnico di Milano, Milan, Italy; Birmingham Medical School, University of Birmingham, Birmingham, UK

**Keywords:** Chest wall motion, Diaphragmatic paralysis, Plication, Optoelectronic Plethysmography

## Abstract

**Background:**

Following diaphragmatic plication for unilateral paralysis, the effect on global chest wall function are unknown. Our hypothesis was that chest wall function would improve in both sides of the chest after plication of the paralysed side.

**Case Presentation:**

Using Optoelectronic Plethysmography, total and regional chest wall volumes were measured in one patient before and after left diaphragmatic plication. Volumes were recorded at quiet breathing.

Respiratory capacity improved during quiet breathing when measured before and 6 months after surgery. These improvements occur at the abdominal-rib cage level in both operated and contralateral. Prior to surgery the abdominal rib cage motion was out of phase to the upper rib cage and abdominal compartment in both sides of the chest. Synchrony of all three compartments was restored after plication.

**Conclusion:**

This physiological study is the first published data in humans to show improvement in chest wall motion both in operated and contralateral side following diaphragmatic plication for unilateral paralysis.

## Background

Diaphragmatic paralysis has a significant symptom burden on patients’ quality of life. This is largely because of its paradoxical movement during respiration [1]. We know that by plicating (double breasting and making taut) the floppy paralysed diaphragm there is improvement in pulmonary function and patients symptoms of breathlessness [2, 3]. However little is known about how this improvement alters dynamic chest wall function. We hypothesize that due to the paradoxical movement of unilateral paralysed diaphragm, abdominal (lower) rib cage chest wall motion on both sides of the chest is impaired and that plication reverses this impairment by preventing that paradoxical movement.

## Case presentation

**Fig. 1:**
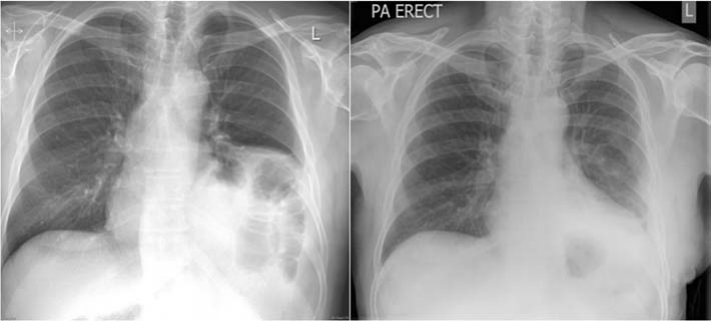
Chest X-rays of the patient before and after surgery. These two PA erect chest radiographs illustrate the thin over-stretched paralysed diaphragm pre-operatively on the *left* which assumes a ‘normal’ position one week following left diaphragmatic plication (*right*)

A prospective observational study was undertaken in a regional thoracic centre in a Local Research Ethics Committee approved study. Informed consent was obtained from the participant. This study was carried out in accordance with The Code of Ethics of the World Medical Association (Declaration of Helsinki) for experiments involving humans. Using Optoelectronic Plethysmography (OEP) [4, 5], total and regional chest wall volumes were measured in a male patient with left sided idiopathic diaphragmatic paralysis (Fig. 1). He was diagnosed with ‘asthma’ and had a long history postural shortness of breath and recurrent respiratory tract infections. He was scheduled for diaphragmatic plication. Surgery was performed under general anaesthesia and through an open incision in the chest or posterolateral thoracotomy. Plication was performed by a double-breasted technique using first interrupted mattress sutures and then a continuous stitch. [2] The patient returned to a dedicated thoracic surgery ward post-operatively. He was managed according to the unit protocol with a daily physiotherapy programme, which included, deep breathing exercises, supported coughing and mobilisation. The patient’s data that was collected and included participants age, height, weight, smoking status and relevant past medical history. Spirometry was performed (Carefusion Microlab) prior to every of chest wall motion capture. Chest wall motion was measured during quiet breathing preoperatively and postoperatively at 6 months. Acquisition of data was performed using SMART suite software [4, 5]. OEP cameras were calibrated each day prior to the tests. The acquisition/procedure required for 79 hemispherical and 10 spherical reflective markers to be placed onto the participants’ chest wall and back using biadhesive hypoallergenic tape [4, 5]. Standard placement of markers according to Cala et al. [6]. OEP acquisition protocol included observations of heart rate, oxygen saturations, blood pressure pre-procedure were recorded from 1 min before quiet breathing to 5 min after the acquisition. All acquisition were done while the patient was in an erect position. During the OEP acquisition, rib cage and abdominal volumes were recorded by eight infrared cameras, operating at 60Hz, tracking the displacement of the markers during vital capacity, quiet breathing for 3 min. From total and compartmental (rib cage and abdominal) chest wall volumes acquired by OEP, the following parameters were obtained: tidal volume (VT), as the total chest wall volume variation, total and compartmental volumes, respiratory rate (RR) and minute ventilation (VT x RR) [4, 5].

Data are presented as mean (±SD) unless otherwise stated. Paired Student t tests were used to compare data before and after surgery for this patient. A statistical significance of 0.05 was used for all analyses.

Our results showed that our patient’s spirometry pre-operatively was impaired. His forced expiratory volume in one second (FEV^1^) was 1.8 l (55 % predicted- patient’s height is 179 cm) and forced vital capacity (FVC) was 2.6 l (62 % predicted). There was a significant improvement in FEV^1^ 6 months after the operation 2.1 l (63 % predicted) compared to the preoperative value (*P* <0.05), similarly there was a significant improvement in (FVC) 3.1 l (73 % predicted) in the same time frame (*P* <0.05).

In quiet breathing the tidal volume improved from 0.65 ± 0.14 L pre-operatively to 0.83 ± 0.10 L 6 months after the surgery (*p* <0.05). Sub-compartment analysis reflects an increase in the contribution of the rib cage abdominal compartment from 0.01 ± 0.02 pre operatively to 0.16 ± 0.03 at 6 months (*p* <0.05) at rest. However the upper rib cage contribution to total tidal volume did not change significantly (Table 1). There is an improvement in the tidal volume in both the operated paralysed side (left) and the contralateral ‘normal’ side. On the operated side the overall tidal volume increases from 0.27 ± 0.05 L preoperatively to 0.29 ± 0.03 L 6 months after surgery during quiet breathing (*p* value <0.05). On the contralateral ‘normal’ side there is also a marked improvement in overall tidal volume from 0.28 ± 0.06 L preoperatively to 0.30 ± 0.05 L 6 months after surgery during quiet breathing (*p* <0.05). These increases in both sides are due mainly to significant improvements in the expansion of the rib cage abdominal compartments (Table 1).

**Table 1:** Respiratory parameters before and after surgery. This table shows the respiratory parameters measured using OEP during quiet breathing before and 6 months after unilateral diaphragmatic plication

	Before operation		6 months		
	Mean	SD	Mean	SD	*P* value
Overall tidal volume (L)	0.65	0.14	0.83	0.1	<0.05
Tidal volume on the operated side (L)	0.31	0.07	0.42	0.05	<0.05
Tidal volume on the non operated side (L)	0.34	0.08	0.42	0.05	<0.05
Minute ventilation (L)	11.2	2.11	13.24	2.01	<0.05
Mean inspiratory flow (L/Sec)	0.49	0.11	0.64	0.03	<0.05
Mean expiratory flow (L/Sec)	0.31	0.07	0.34	0.07	0.11
Overall pulmonary rib cage tidal volume (L)	0.1	0.04	0.08	0.02	0.45
Overall abdominal rib cage tidal volume (L)	0.01	0.02	0.16	0.03	<0.05
Overall abdominal tidal volume (L)	0.55	0.11	0.59	0.08	0.06
Pulmonary rib cage tidal volume – non-operated side (L)	0.05	0.02	0.06	0.01	0.06
Pulmonary rib cage tidal volume – operated side (L)	0.04	0.02	0.02	0.01	0.1
Abdominal rib cage tidal volume – non- operated side (L)	0.01	0.01	0.06	0.02	<0.05
Abdominal rib cage tidal volume – operated side (L)	0	0.01	0.1	0.02	<0.05
Abdominal tidal volume – non-operated side (L)	0.28	0.06	0.3	0.05	0.08
Abdominal tidal volume – operated side (L)	0.27	0.05	0.29	0.03	<0.05

**Fig. 2:**
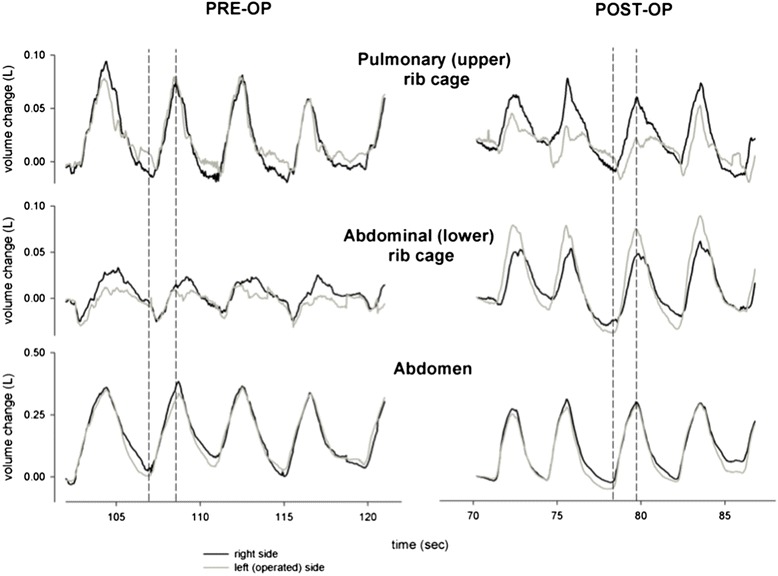
Synchrony between chest compartments. The figure shows the phases of breathing on both sides of the chest wall during quiet breathing before the operation and 6 months after unilateral diaphragmatic plication. We found that before the operation, the expansion of the abdominal rib cage was lagging behind the abdominal expansion, showing complete inward paradoxical movement during inspiration in both sides of the chest. After the operation that inward paradoxical movement disappeared in both sides, and resulted in complete synchronization of both sides following unilateral diaphragmatic plication

We found that during quiet breathing before the operation, the expansion of the abdominal rib cage was lagging behind the abdominal expansion during inspiration in both sides of the chest. After the operation this lag disappeared in both sides, and resulted in complete synchronization of both sides following unilateral diaphragmatic plication (Fig. 2).

Our study shows that lung function as assessed by spirometry improves at 6 months post operatively compared to pre-operative values in a patient who has undergone surgical plication for unilateral diaphragmatic paralysis. This is in keeping with other large published results. Feeman et al. have also reported improvement spirometry, dyspnoea score and functional status in 41 patients who had diaphragmatic plication at 6 months after the operation [2]. He reported improvement in FEV1 and FVC of 21 and 17 % respectively, which is comparable to our results, as we observed improvements in FEV1 and FVC of 18 and 24 % respectively during the same time period. These improvements seem to be sustained long term as reported by Celik et al. who observed in 13 patients who had undergone diaphragmatic plication with a mean follow of 5.4 years an improvement in FVC and FEV1 of 43.6 ± 30.6 % (*p* < 0.05) and 27.3 ± 10.9 % (*p* < 0.05) respectively [3].

Paralysis of the diaphragm results in a paradoxical motion of both sides of the lower rib cage with inspiration which results in severe respiratory impairment than if the diaphragm was fixed in position [1]. We hypothesized that by abolishing the superior paradoxical movement of diaphragm by plication, this will improve the efficacy of the respiratory muscles, which will results in improved lung volumes. Our results support our hypothesis; in our patient there was an 28 % improvement during quiet breathing in the overall tidal volume of the chest cavity 6 months after the surgery. This is a direct result of preventing the paradoxical movement of the paralyzed diaphragms. We also found during quiet breathing the increase of tidal volume 6 months after surgery was mainly due to an increase of the contribution of rib cage abdominal compartment of the chest wall. Surprisingly the magnitude of improvement is similar in both operated and contralateral side 35 and 23 % respectively. In an animal model Takeda et al. reported improvement in the contralateral hemi-diaphragm contractility and diaphragmatic contribution to breathing after diaphragmatic plication following unilateral diaphragmatic paralysis in eight dogs [6]. Takeda’s results have not been replicated in humans. Celik et al. reported using fluoroscopy that the plicated diaphragm was immobile, remained elevated and had no paradoxical motion but did not comment on function of the diaphragm on the non operated side [3]. The notion that the contra-lateral side is so badly impaired by unilateral paralysis and improves so dramatically after surgery has never been reported and leads us to publish this data despite it being in only one patient. From a physiological and anatomical viewpoint both sides of the diaphragm are interconnected. The central tendon acts as a rigid mechanical linkage between the two sides. Simplistically one can envisage when the over-stretched paralysed side loses tension then the interconnected ‘normal side’ has nothing to contract against. Plication restores this tension even static and thus restores function of the contralateral diaphragm. By this mechanism it gets rid of the paradoxical motion of the diaphragm and restores synchrony in the interconnected chest compartments on both sides of the chest.

## Conclusion

In summary this study is the first published data in humans to show almost equal improvements in dynamic chest wall motion/volumes both in operated and contra-lateral side following plication for idiopathic unilateral diaphragmatic paralysis.

### Consent

Written informed consent was obtained from the patient for publication of this Case report and any accompanying images. A copy of the written consent is available for review by the Editor-in-Chief of this journal.

## Abbreviations

OEP: Optoelectronic Plethysmography
VT: tidal volume
RR: respiratory rate
FEV^1^: forced expiratory volume in one second
FVC: forced vital capacity
PA: posterior-anterior
L: liter
Pre-op: before surgery
Post-op: after surgery
